# VIM-1 Metallo-β-lactamase in *Acinetobacter baumannii*

**DOI:** 10.3201/eid1206.051097

**Published:** 2006-06

**Authors:** Athanassios Tsakris, Alexandros Ikonomidis, Spyros Pournaras, Leonidas S. Tzouvelekis, Danai Sofianou, Nicholas J. Legakis, Antonios N. Maniatis

**Affiliations:** * University of Athens, Athens, Greece;; † University of Thessalia, Larissa, Greece;; ‡ Hippokration University Hospital, Thessaloniki, Greece

**Keywords:** Acinetobacter baumannii, VIM-1, OXA-51, OXA-58, carbapenemase, imipenem, meropenem, dispatch

## Abstract

In 2004 and 2005, 5 metallo-β-lactamase (MBL)-positive *Acinetobacter baumannii* isolates were found in 2 Greek hospitals. Isolates were unrelated and carried *bla*_VIM-1_ in a class 1 integron; *bla*_OXA-51-_ and *bla*_OXA-58-like_ carbapenemase genes were also detected. VIM-1 MBL in *Acinetobacter* spp. causes concern, given the increasing resistance of this species.

In the last few years, resistance to antibacterial drugs has been increasing in *Acinetobacter* spp., which will likely become a substantial treatment challenge in the future (*1*). Carbapenems have potent activity against *Acinetobacter* spp. and are usually the drugs of choice against multidrug-resistant *Acinetobacter baumannii* isolates. *Acinetobacter* spp. may develop resistance to carbapenems through various mechanisms, including class B and D carbapenemase production, decreased permeability, altered penicillin-binding proteins, and rarely, overexpression of efflux pumps (*2**,**3*).

In Europe, carbapenem resistance in *A. baumannii* has been sporadically attributed to the production of IMP-type metallo-β-lactamases (MBLs) and OXA-type carbapenemases (*4*). VIM-2–producing *Acinetobacter* spp. have been isolated in the Far East (*4**,**5*) and on 1 occasion in Germany (*6*). In this study, we report the appearance of the VIM-1 MBL determinant among *A. baumannii* in Greece.

## The Study

We included in the study *A. baumannii* clinical isolates from tertiary care hospitals in 2 different Greek regions (Hippokration University Hospital, Thessaloniki, and University Hospital of Larissa, Thessalia) that were positive by the imipenem-EDTA double-disk synergy test (DDST) from March 2004 to March 2005. Bacteria were provisionally identified to the genus level by the Vitek 2 automated system (bioMérieux, Marcy l'Étoile, France) and the ATB 32GN system (bioMérieux). Antimicrobial drug susceptibility testing of the DDST-positive isolates for β-lactams (aztreonam, ceftazidime, cefepime, imipenem, meropenem, and piperacillin), β-lactam/β-lactamase inhibitor combinations (ampicillin/sulbactam, piperacillin/tazobactam), aminoglycosides (amikacin, gentamicin, netilmicin, and tobramycin), fluoroquinolones (ciprofloxacin and ofloxacin), and colistin was performed by Etest and Etest MBL (AB Biodisk, Solna, Sweden). The Clinical and Laboratory Standards Institute (CLSI) interpretative criteria were used (*7*), and *Pseudomonas aeruginosa* ATCC 27853 was used as control.

Polymerase chain reaction (PCR) testing of the synergy-positive isolates for carbapenemase genes was done by using consensus primers for *bla*_IMP_ (*8*), *bla*_VIM_ (*9*), *bla*_SPM_ (*10*), *bla*_OXA-23-like_ (*11*), *bla*_OXA-24-like_ (*11*), *bla*_OXA-58-like_ (*11*), and *bla*_OXA-51-like_ (*12*). Pulsed-field gel electrophoresis (PFGE) of *Apa*I-digested genomic DNA was performed in the *bla*_VIM-1_-positive isolates, and the banding patterns were compared by using criteria proposed by Tenover et al. (*13*). The potential for conjugational transfer of imipenem resistance was examined in filter matings by using *Escherichia coli* 20R764 (*lac*^+^ rif^r^) as the recipient. Donor and recipient were mixed in a 1:5 ratio, and transconjugants were selected on MacConkey agar plates containing 100 μg/mL rifampicin and imipenem at concentrations of 0.5 to 2 μg/mL or 2 μg/mL ceftazidime.

Five *A. baumannii* clinical isolates that were MBL producers on the basis of DDST were detected among collections of isolates from patients hospitalized during the study period. Two of the isolates were recovered from blood cultures, 1 from bronchial secretions, 1 from a urine specimen, and 1 from cerebrospinal fluid; 2 isolates that had reduced susceptibility to carbapenems were not positive by the Etest MBL (Table). Imipenem MICs ranged from 4 to >32 μg/mL, while meropenem MICs were 2–32 μg/mL. *P. aeruginosa* ATCC 27853 was consistently characterized as having imipenem MIC of 2 μg/mL and meropenem MIC of 0.5 μg/mL. The 5 *A. baumannii* isolates were multidrug resistant; they showed resistance to all other antimicrobial drugs tested, with the exception of colistin.

**Table:** Characteristics of *bla*_VIM-1_-bearing *Acinetobacter baumannii* isolates*†

Isolate no.	Region of isolation	Material	PFGE type	Etest IMP MIC (μg/mL)	Etest MBL (IMP+EDTA)	Etest MER MIC (μg/mL)	*bla*_OXA-51-like_ status	*bla*_OXA-58_ status
1	Thessaloniki	Blood	Ia	4	<1	2	+	–
2	Thessalia	Bronchial fluid	II	32	2	4	+	+
3	Thessalia	CSF	III	4	<1	2	–	–
4	Thessalia	Blood	IV	>32	1	32	+	–
5	Thessaloniki	Urine	Ib	8	<1	4	+	+

*The 5 isolates exhibited resistance to all alternative antimicrobial drugs except colistin. †PFGE, pulsed-field gel electrophoresis; IMP, imipenem; MBL, metallo-β-lactamase; MER, meropenem; CSF, cerebrospinal fluid.

We did not detect *bla*_IMP_, *bla*_SPM_, *bla*_OXA-23-like_, or *bla*_OXA-24-like_ in any of the 5 isolates, whereas *bla*_VIM_ was detected in all of them. In 2 isolates, *bla*_OXA-51-_ and *bla*_OXA-58-like_ genes were also simultaneously present, while 2 more carried a *bla*_OXA-51-like_ gene (Table). By sequencing both strands of the entire *bla*_VIM_ amplicons (*14*), a *bla*_VIM-1_ sequence identical to that available in the database was identified. Sequencing *bla*_OXA-51-like_ amplicons identified *bla*_OXA-66_ in all cases, while *bla*_OXA-58-like_ alleles were classical *bla*_OXA-58_ in both cases. In 2 isolates, 1 from each region, PCR mapping of the integron that possibly carried *bla*_VIM-1_, with primers 5´ CS and a set of primers for genes *aacA*, *dhfrI*, *aadA*, *qacE*Δ1, and *sul*, showed a class 1 integron with a variable region including from 5´ to 3´ *bla*_VIM-1_, *aacA7*, *dhfr*I, and *aadA1* gene cassettes. Sequencing of the overlapping PCR amplicons showed that this class 1 integron contained the *intI1* gene with a strong P1 promoter, an inactivated (without a GGG insertion) P2 promoter, an *attI1* site, and the *bla*_VIM-1_ gene cassette with its 59-base element identical to those reported previously in other gram-negative bacteria from Greece (*15*). PFGE showed that the 5 *bla*_VIM-1_-positive isolates did not form a genetically homogeneous group; they belonged to 4 distinct types. The 2 isolates from Thessaloniki were subtypes of the same clone (Table, Figure). In none of the 5 isolates was *bla*_VIM-1_ transferable to the susceptible *E. coli* host after repeated conjugal experiments with imipenem or ceftazidime selection. The 5,387-bp nucleotide sequence of the integron structure reported in this study has been submitted to the EMBL/GenBank/DDBJ sequence databases and has been assigned accession no. DQ112355.

**Figure:**
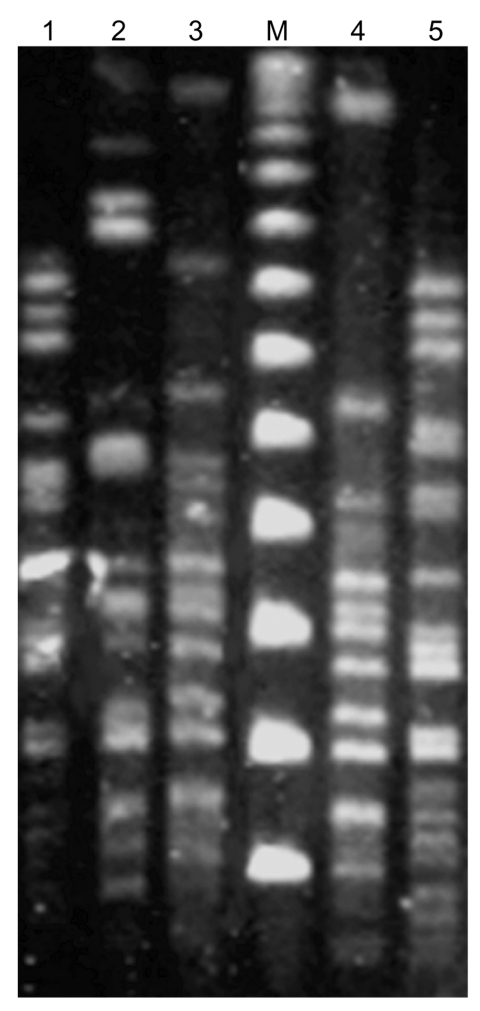
Pulsed-field gel electrophoresis of *Apa*I-restricted genomic DNA of the 5 *bla*_VIM-1_-positive *Acinetobacter baumannii* isolates. The index numbers of the isolates are those listed in the Table. Lane M, molecular mass marker (48.5 kb).

## Conclusions

This is the first report of the VIM-1 determinant in *A. baumannii* in the world. Occurrence of VIM-2 MBL among *A. baumannii* and *Acinetobacter* genomospecies 3 isolates has previously been described among clinical isolates in Korea (*4**,**5*). In Europe, IMP-type enzymes had been reported in single *A. baumannii* isolates from some European regions (*4*) and VIM-2 from 1 German hospital (*6*), while MBLs have not been detected in *A. baumannii* from the United States despite carbapenem resistance there (*1*). Though anticipated because of the circulation of *bla*_VIM_ genes in several other gram-negative species in Europe and the ability of *Acinetobacter* spp. to acquire foreign DNA, this evolution is worrisome.

Retention of moderate susceptibility to carbapenems by *bla*_VIM_-positive *A. baumannii* isolates in our study may seem unexpected, since MBLs hydrolyze these compounds. However, MBL production in gram-negative bacteria may not substantially increase carbapenem MICs without the simultaneous operation of other mechanisms, such as impaired permeability (*2**,**4*). Furthermore, 2 of the 4 strains carrying oxacillinase genes with carbapenemase properties had imipenem MICs not higher than the CLSI breakpoints for resistance. Recently, *bla*_OXA-51-like_ genes have been shown to be possibly naturally occurring (*12*), while OXA-58 enzymes play a minor role in carbapenem resistance in *A. baumannii*, and strong promoter sequences are needed for higher levels of resistance to carbapenems (*2*).

During the last few years, *A. baumannii* has been increasingly isolated from severely ill patients, and its usual cross-resistance to most available antimicrobial drugs, including carbapenems, poses substantial problems worldwide and especially in the United States (*1*). In New York approximately two thirds of isolates are carbapenem resistant (*3*). In our region, *Acinetobacter* spp. are frequent nosocomial pathogens and are commonly multidrug resistant, which leads to the extensive use of carbapenems and, lately, polymyxins. The presence of MBLs among carbapenem-resistant *Acinetobacter* spp. from different Greek regions emphasizes the need for restricted use of carbapenems and early recognition of strains producing these enzymes. Although Etest MBL was reliable to detect VIM-2-producing *Acinetobacter* spp. in Korea (*5*), the assay seems unable to identify MBL-positive isolates exhibiting relatively low carbapenem MICs. Therefore, our diagnostic laboratories should screen *Acinetobacter* spp. with imipenem-EDTA DDST or alternative DDSTs, such as those using 2-mercaptopropionic acid, which appears to be more sensitive for detecting MBLs among *Acinetobacter* spp (*4*). Whether carbapenems might be appropriate to treat infections with low-level carbapenem-resistant or susceptible *bla*_VIM_-bearing *A. baumannii* isolates has yet to be determined.
